# Antioxidant Potential of Xanthohumol in Disease Prevention: Evidence from Human and Animal Studies

**DOI:** 10.3390/antiox13121559

**Published:** 2024-12-19

**Authors:** Jakub Piekara, Dorota Piasecka-Kwiatkowska

**Affiliations:** Department of Food Biochemistry and Analysis, Poznan University of Life Sciences, Mazowiecka 48, 60-623 Poznan, Poland; jakub.piekara@up.poznan.pl

**Keywords:** xanthohumol, hop, phenolic compound, disease, supplementation

## Abstract

Xanthohumol (XN) is a phenolic compound found in the largest amount in the flowers of the hop plant, but also in the leaves and possibly in the stalks, which is successfully added to dietary supplements and cosmetics. XN is known as a potent antioxidant compound, which, according to current research, has the potential to prevent and inhibit the development of diseases, i.e., cancer and neurodegenerative diseases. The review aims to examine the antioxidant role of XN in disease prevention, with an emphasis on the benefits and risks associated with its supplementation. The regulation by XN of the Nrf2/NF-kB/mTOR/AKT (Nuclear factor erythroid 2-related factor 2/Nuclear factor kappa-light-chain-enhancer of activated B cells/Mammalian target of rapamycin/Protein Kinase B) pathways induce a strong antioxidant and anti-inflammatory effect, among others the acceleration of autophagy through increased synthesis of Bcl-2 (B-cell lymphoma 2) proteins, inhibition of the synthesis of VEGF (Vascular-endothelial growth factor) responsible for angiogenesis and phosphorylation of HKII (Hexokinase II). It is the key function of XN to ameliorate inflammation and to promote the healing process in organs. However, existing data also indicate that XN may have adverse effects in certain diseases, such as advanced prostate cancer, where it activates the AMPK (activated protein kinase) pathway responsible for restoring cellular energy balance. This potential risk may explain why XN has not been classified as a therapeutic drug so far and proves that further research is needed to determine the effectiveness of XN against selected disease entities at a given stage of the disease.

## 1. Introduction

Xanthohumol (XN) and its derivatives Isoxanthohumol (IXN), 8-prenylnaryngenin (8-PN), and 6-prenylnaryngenin (6-PN) belong to the group of flavonoids, naturally occurring and synthesized in plant cells with a particularly high abundance in common hops (*Humulus lupulus* L.). Flavonoids, including XN and its derivatives, play an essential role in proper plant development, being their first line of defense against environmental stressors, insects, and herbivores. These compounds affect the formation of unpleasant odors and tastes, which protects them from pests and neutralizes reactive oxygen species (ROS) through antioxidant action. The study of these plant defense mechanisms, which enable certain plants to thrive despite unfavorable environmental conditions, has drawn scientific attention to their inherent resistance. This, in turn, has sparked interest in the therapeutic potential of phenolic compounds, particularly considering the role oxidative stress plays in many human diseases [1,2,3,4].

The structure of XN, classified as a prenylated flavonoid, was identified in 1957, and research into its application has been underway for about four decades, gaining importance, especially in the cosmetics industry and the production of supplements. XN’s antioxidant activity has garnered significant attention due to its ability to scavenge free radicals, reduce oxidative stress, and modulate inflammatory pathways [5]. These properties are highly relevant in the prevention of chronic diseases, such as intestine disease, metabolic disorders, and neurodegenerative conditions, which are often driven by oxidative damage and inflammation. Furthermore, animal and human studies have demonstrated the effectiveness of XN supplementation in improving health parameters, both when applied topically (e.g., in creams) and taken orally as part of dietary supplements.

XN is abundant in hops, especially in the cones of this plant. Female hop inflorescences, used in the production of beer, are characterized by a high content of phenolic compounds, which give beer the characteristic bitterness desired by consumers. While traditionally considered waste, hop sludge (hot trub) and other by-products of brewing are now recognized as valuable sources of bioactive compounds, including XN and its derivatives [6]. However, thanks to scientific advances and a growing awareness of the need to implement a closed-loop economy, both the management of hot decoction and post-production waste from hop plantations have gained valuable new applications [7,8]. Despite the observed increase in applications, it seems that recovered XN could find wider use and be an ingredient in dietary supplements for the prevention of diseases.

The aim of this study is to review current scientific evidence on the antioxidant functions of XN and its metabolites, focusing on their effectiveness as supplements in human and animal models. By consolidating findings from various studies, we seek to clarify the potential of XN in preventing oxidative stress-related diseases and enhancing overall human health.

## 2. XN and AMPK Pathway in Digestive System Disease Prevention

### 2.1. Enhancement of AMPK Phosphorylation and Antioxidative Pathways by XN

AMPK (activated protein kinase) is a protein kinase activated by *Thr172* phosphorylation (Phospho-AMPK-alpha), which can be increased by XN. AMPK protein kinase is a basic metabolic pathway that restores energy balance at the cellular and physiological level in response to metabolic stress related to the proper functioning of the body and daily activities. A properly functioning AMPK metabolic pathway is a kind of mechanism that rearranges weights in the form of metabolic products on the beam of the anti-weight swing, activating catabolic processes (obtaining ATP) while inhibiting anabolic processes (ATP breakdown) and vice versa, providing cells with energy and building and energy components [9].

**Figure 1:**
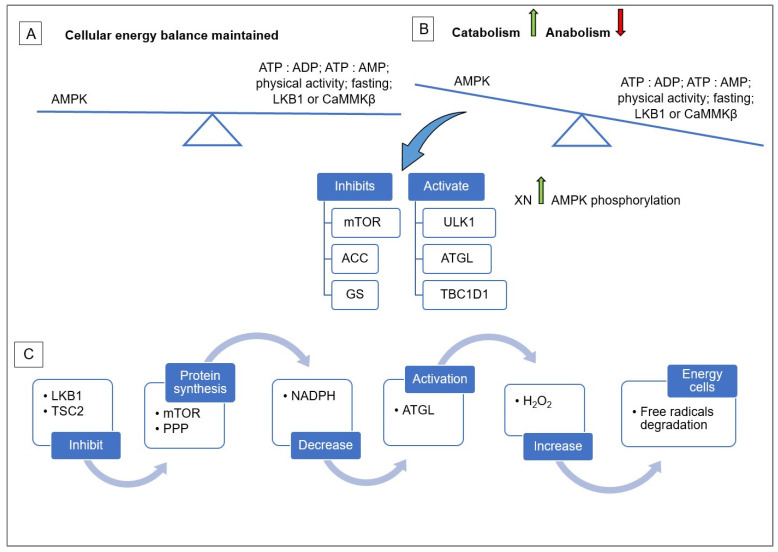
The scheme of AMPK pathway of cellular energy balance: maintained (**A**), compromise (**B**), and recovery (**C**). Arrows show the effects: increase 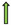, decrease 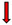. mTOR: mammalian target of rapamycin; ACC: Acetyl-CoA carboxylase; GS: glycogen synthase; ULK1: unc-51 like autophagy activating kinase 1; ATGL: Adipose triglyceride lipase; *TBC1D1*: TBC1 domain family member 1; *TSC2*: Tuberous sclerosis complex 2; PPP: Pentose phosphate pathway; NADPH: Nicotinamide adenine dinucleotide phosphate; H_2_O_2_: Hydrogen peroxide.

AMPK is involved in the metabolism of macronutrients in eukaryotic cells. It is a kind of sensor of changes in the ratio of ATP (adenosine triphosphate) to ADP (adenosine diphosphate) and is not activated when the concentration of ATP is higher than ADP. During oxidative stress, the changes that occur in the cells contribute to the decrease in ATP, and thus, the initialization of AMPK occurs, which returns to the baseline state when the ratios of ATP to ADP and ATP to AMP (adenosine monophosphate) are established. The AMPK pathway process is presented in Figure 1. In the case of XN supplementation, this process is accelerated, protecting cells from free radicals. There are two ways of restoring cellular homeostasis: through catabolism with the production of ATP molecules and anabolism with the involvement of mTORC1 (mammalian target of rapamycin complex 1). AMPK has three different regulatory catalytic subunits: α, β, and γ. It is a protein kinase activated by phosphorylation of a threonine residue occurring in the domain of the alpha catalytic subunit, along with it the additional subunits STRAD (Ste20-related adaptor protein α) and a protein kinase that suppresses the development of cancer cells LKB1 (liver kinase B1), which is activated even before AMPK. AMPK contains three gamma subunits that are associated with ATP: ADP, ATP:AMP. Importantly, AMPK activation during XN supplementation does not require kinases, as phosphorylation because of supplementation is carried out on its own [10,11].

The second known pathway for AMPK activation is CaMKK-α (Calcium/calmodulin-dependent protein kinase kinase α) and CaMKK-β (Calcium/calmodulin-dependent protein kinase kinase β), which are activated by many ligands on the cell membrane. Activation, primarily of the CaMKK-β isoform, requires the accumulation of a sufficiently high concentration of calcium ions inside the cell, which is not always desirable [12]. This pathway is of particular importance in the development of cancer in men.

### 2.2. Antioxidative Proprieties of XN and Enhanced AMPK Phosphorylation: Risks and Benefits

The enzymes that are part of AMPK contribute to the maintenance of intracellular homeostasis. From a holistic point of view, it is necessary to distinguish cancer cells from other cells and to determine the effect of XN supplementation on the growth and proliferation of unwanted cells. AMPK does not act selectively; it also contributes to the survival of cancer cells by providing energy from glycolysis and removing unnecessary metabolic products.

The task of AMPK is to maintain cellular homeostasis by regulating the process of autophagy, immunomodulation, glycolysis, integration of hormones in metabolic processes, and protein synthesis [13,14,15,16]. The process of autophagy, which involves the degradation of cytoplasmic structures, is activated during the period of insufficient energy supply to maintain the homeostasis of cell metabolism. The role of AMPK in this process is to increase the activity of ULK1 kinase, initiating the degradation process by inhibiting the regulator of protein synthesis mTORC1 and increasing the activity of *TSC2*. In such cases, cells, regardless of their type, take the energy necessary for further development also by inhibiting GS and activating *TBC1D1*, which increases glucose uptake by cells [9,17].

Activating autophagy processes to fight cancer cells may prove to be a double-edged sword. There is a lot of scientific evidence that the biological effect of chemotherapy can be increased along with progressive autophagy. However, e.g., in cases such as prostate cancer and malignant tumors, this process enhances the growth of cancer cells. Conversely, direct phosphorylation of UKL1 by mTORC1, as well as the direct and indirect ATG13 (Autophagy-related protein 13), stops the autophagy process, which inhibits the growth of cancer cells. Cancerous tumors, even without progressive angiogenesis, show the ability to communicate with other cells through metabolic stress resulting from the lack of access to nutrients, which is why the process of autophagy is a way for them to survive and then proliferate. During a deficiency of energy compounds in cells, LKB1 contributes to the activation of AMPK due to low concentrations of ATP and high concentrations of ADP and AMP. Then, the PPP pathway from which NADPH is formed, which in turn leads to an increased concentration of H_2_O_2_, which degrades free radicals through increased activation of ATGL due to the inhibition of ACC, providing the cell with energy primarily in the case of prostate cancer and glioma, which are also dependent on the concentration of calcium ions activating the CaMKK2-AMPK axis (Calcium/calmodulin-dependent kinase kinase protein 2–activated protein kinase axis) [18,19,20,21,22]. Therefore, the question should be asked whether XN, by indirectly affecting the activation of AMPK, does not contribute to the growth of tumors. However, there is no clear evidence so far because most studies in this field, such as the studies on gastrointestinal cancers described in the laterpart of the paper, are conducted on rodents. The disadvantage of such studies is the limited time range, which does not allow observations on the effect of XN on cancer cells at a critical stage.

### 2.3. XN Stimulation of Gastric Mucosa Healing Process

The stomach is one of the first organs that come into contact with chewed food. The primary function of the stomach in the digestive system is the initial degradation of food into smaller molecules. This applies primarily to proteins and fats. In addition to the initial digestive process, it plays a protective role for the rest of the digestive tract and then the body against microorganisms. The prevailing low pH value is an effective protection against pathogens due to the hydrochloric acid secreted by the gastric mucosa. Unfortunately, some pathogens, in particular *Helicobacter pylori*, due to their enzymatic apparatus, locally neutralize the low pH of gastric juice, posing a health risk and causing ulcers.

The specific, helical structure of *H. pylori* allows it to literally penetrate the gastric mucosa, which, because of damage by the pathogen, regenerates in response to inflammation. Chronic inflammation of the gastric mucosa is the main cause of the formation of polyps of this organ, which, over time, can develop into a malignant tumor, and the prognosis of recovery leaves no illusions. Currently, the literature does not allow for an unambiguous statement of the effect of XN and its metabolites on lesions within the stomach. However, the results of studies using ethanolic extracts from hop leaves and cones in which XN is found suggest its therapeutic effect. Minaiyan et al. [23] found that the use of hop leaf and cone extract at doses of 100 and 150 mg/kg body weight in the treatment of gastric ulcers in rats resulted in their reduction. Wei et al. [24], in an in vitro study of gastric cancer cell changes, found that XN isolated from hops effectively and selectively inhibits the growth and proliferation of cancer cells. 

### 2.4. Reduction of ROS and Inflammation in Pancreatic Diseases via NFr2/ARE/HO-1 Pathway Activation by XN

The pancreas is an exocrine and endocrine organ belonging to the digestive and endocrine systems. Pancreatic juice, which is supplied through the pancreatic duct common to the lumen of the duodenum, contains numerous hydrolytic enzymes important in the process of catabolism of nutrients, proteins, fats, and saccharides. In addition, this organ produces insulin through beta cells, which is one of the most anabolic hormones that regulate blood glucose levels through its active transport to cells, while in the case of nutrient deficit or low blood glucose levels, it proceeds to synthesize alpha glucagon in cells an antagonistic hormone that increases blood glucose levels [25,26,27]. Pancreatic diseases, such as acute and chronic inflammation and cancer, often manifest themselves in the form of intestinal problems caused by the lack of synthesis of adequate amounts of digestive enzymes, which means that the consumed food cannot be digested properly, which in turn contributes to weight loss due to lack of nutrients [28]. AP (acute pancreatitis) is a life-threatening condition in which, among other things, necrosis of the organtissues occurs. So far, no effective treatment has been developed. This condition may be the result of metabolic syndrome, excessive alcohol consumption, and gallstones blinding the pancreatic juice outlet [29,30]. If the cause of AP is gallstones, they are removed, restoring the proper functioning of the organ in a short time. On the other hand, the postoperative effects of pancreatic resection, in the form of organ failure to produce an adequate number of digestive enzymes, may accompany patients for the rest of their lives [31]. A study conducted by Huangfu et al. [32] shows that XN reduces the expression of proteins responsible for causing inflammation by lowering the cytokines TNF-α (Tumor necrosis factor-α), IL-6 (Interleukin 6), IL-17 (Interleukin 17), with a simultaneous increased synthesis of IL-10 (Interleukin 10) responsible for reducing inflammation. In addition, XN has been shown to have the ability to reduce ROS, the bearing of which develops in AP, through the activation of Nrf2 (Nuclear factor erythroid 2-related factor 2), which consists largely in the transition from the cytosol to the cell nucleus. Nrf2 attaches to ARE (Antioxidant responsive element) and initiates transcription of among others HO-1 (Heme oxygenase 1), which has anti-inflammatory activity in the pancreas [33]. Hangfu et al. also demonstrated XN’s ability to lower concentrations of the mTOR and Protein Kinase B (AKT) proteins. Inhibition of this pathway allowed to increase the concentration of autophagy, whose task, among others, is to degrade ROS (Reactive oxygen species) and prevent the development of cancer within inflammation [34].

Treatment of such an important organ as the pancreas requires a global view of the problem. Yang et al. [35] proved in a study on mice that inhibition of the autophagy process contributes to the rapid reduction of the cancerous tumor. This is because the increased process of autophagy in the region of cancer cells can contribute to their growth by reducing oxidative stress caused by cancer cells, which makes it easier for them to provide the nutrients necessary for growth [36]. On the other hand, in order for cancer cells to develop effectively, developing in the initial stage without access to oxygen and nutrients, angiogenesis must occur. Angiogenesis and proliferation of pancreatic cancer cells are closely related to NF-kB (Nuclear factor kappa-light-chain-enhancer of activated B cells), which affects the expression of genes that synthesize pro-inflammatory cytokines and stimulate cell proliferation and proteins that prevent apoptosis. Saito et al. [37], in a study conducted on mice with deliberately developed pancreatic cancer as well as on cells line, proved that XN, by inhibiting NF-kB, contributes to the reduced VEGF (Vascular-endothelial growth factor) and IL-8 (Interleukin 8) responsible for angiogenesis on the level of mRNA and pancreatic cancer cells. In addition, the tumor studied had lower levels of Ki-67 (proliferation marker) and CD31 (Cluster of differentiation 31), which was associated with a reduction in cancer compared to the control group. An important factor in the process of inhibiting cancer turned out to be the dose of XN. The use of a dose below 5 µmol/l only resulted in the inhibition of angiogenesis by suppressing NF-κB, while the dose of >10 µmol/l additionally inhibited the proliferation of cancer cells [38,39].

### 2.5. Inhibition of Stellate Cell Proliferation by XN Through NF-κB Pathway Suppression

The liver is the largest internal organ of the human body. It plays the role of a center of biochemical transformations, including de novo glucose synthesis, conversion of fatty acids (FA) to smaller subunits, transformation of hormones into biologically active forms and secretory function of bile acids. Since the liver is responsible for the absorption and excretion of metabolites, it is constantly exposed to both toxic compounds entering the body from the external environment and to an unfavorable diet [40,41]. The liver, despite its size and a lot of functions, does not have pain receptors, so diseases such as MASLD (metabolic associated fatty liver diseaseor cirrhosis can develop unnoticed for many years. Often, only laboratory tests or the sensation of pain in the right subcostal arch provide information to the patient about a potential disease [42].

Dyslipidemia is one of the life-threatening factors due to the increased risk of myocardial infarction caused by atherosclerosis. The cause of the disease is primarily an inappropriate nutritional model rich in high-energy and fat-rich foods and a genetic factor associated with the expression of the lipoprotein lipase inhibitor, ANGPTL3 (Angiopoietin-Like Protein 3), contributing to an increase in serum TAG (triglycerides) levels [43].

One of the best models for studying metabolic diseases in humans is zebrafish [44]. A study by Gao et al. [45] using the above-mentioned animal model to compare the efficacy of XN and its metabolites 6-PN (6-prenylnaryngenin) and 8-PN (8-prenylnaryngenin) as inhibitors of ANGPTL-3 synthesis showed that it is XN that has the highest inhibitory activity. The increase in cholesterol and FA metabolism is due to the dual action of XN within liver cells. On the one hand, by acting as an antagonist, it contributes to the reduction in ANGPTL-3 synthesis, and on the other hand, it activates the LXR-α receptor (Liver X receptor α), which, after XN attachment, contributes to increased synthesis of LPL (Lipoprotein lipase) [46,47].

LXR-α, a nuclear receptor, regulates the synthesis of Mylip (Myosin regulatory light chain interacting protein), which inhibits LDL (low-density lipoprotein) receptor activity depending on the concentration of low-density lipoproteins. Chen et al. [48] demonstrated that XN, after attaching to the LXR-α receptor, induces the synthesis of a protein that inhibits the activity of the LDL receptor, which causes a decrease in serum LDL level.

Despite its ability to regenerate, the liver, under the influence of chronic inflammation, produces fibrosis in the areas of damage to a given lobe [49]. The reason for this is damage to hepatocytes resulting from an inappropriate lifestyle, drug abuse, or bacterial and viral infections [50]. Chronic inflammation within hepatocytes leads to excessive production of liver stellate cells, which, depending on their activation, play two roles. In the resting phase, these cells accumulate vitamin A in the cytoplasm in the form of retinol ester, while under the influence of inflammation, they move to the active phase, showing the ability to proliferate and promote the formation of fibrosis within the organ [51,52]. The main factor contributing to the process of fibrosis in the liver is the synthesis of TGF-β1 (Transforming growth factor β) by macrophages, which activates NF-κB in Kupffer cells and hepatocytes in response to inflammation [53]. Transdifferentiation and proliferation of stellate cells to myofibroblasts are then observed, synthesizing, among others, type I collagen, which contributes to liver fibrosis and scarring [54,55]. Fibroblast regression is possible only when inflammation is inhibited; then, stellate cells return to the inactivated phenotype, and as a result, macrophages proceed to synthesize collagenases, hydrolyzing collagen fibrosis, leading to their resorption [40].

Dorn et al. [56], based on studies conducted in mice, demonstrated that XN contributes to the reduction of NF-kB, thereby inhibiting the liver fibrosis process. The decreased concentration of the transcription factor leads to a reduction in the synthesis of MCP-1 (monocyte chemoattractant protein-1) and ICAM-1 (Intercellular Adhesion Molecule 1), further suppressing fibrosis. Similar findings were observed by Kang et al. [57] in a study on mice with MASLD, where XN was shown to inhibit the progression of liver fibrosis.

Another significant cytokine involved in fibrosis of the liver and gastrointestinal tract is TNF-β (Tumor Necrosis factor-beta). Yun-Mi et al. [58] indicated the ability of XN to inhibit the expression of pro-inflammatory genes of the described pathological condition. Cho et al. [59] identified a critical point in fibrosis progression: the covalent bonding between the electrophilic carbon center of α, the β-unsaturated carbonyl moiety XN, and the cysteine residue (Cys 99) in IKKβ (Inhibitor of nuclear factor kappa-B). The created complex prevents the activation of NF-κB, suggesting that XN acts as a competitive inhibitor of pro-inflammatory cytokines, blocking their nuclear translocation and thereby inhibiting the expression of pro-inflammatory genes [60].

### 2.6. Mechanisms of XN in Colorectal Cancer Suppression

The colon is the final section of the digestive tract, with a length of about 1.5 m. The main task of the colon is to form stool from food residues metabolized in the small intestine, thus removing toxic metabolic products with the active participation of the microbiota. On the other hand, it undergoes water and amino acid resorption, synthesis and absorption of B vitamins and vitamin K [61,62,63].

Colorectal cancer is one of the most common life-threatening diseases affecting both women and men. The causes of carcinogenic changes in the colon, in addition to genetic predisposition, are primarily the inappropriate nutritional model associated with the Western lifestyle. Consumption of high-energy foods rich in pro-inflammatory ingredients, including sweetened and alcoholic beverages, can lead to inflammation and intestinal dysbiosis. This, in turn, promotes the growth of bacteria with pro-inflammatory and oncogenic properties, such as *Klebsiella* or *Fusobacterium nucleatum*, which may contribute to the development of colorectal cancer [64,65,66].

While the detection of colorectal cancer at an early stage makes it possible to remove it, even during periodic colonoscopy, and histopathological analysis allows us to determine its malignant nature or not, the detection of malignant tumor at a late stage of development requires treatment with chemotherapy and then surgical treatment [67]. Detection of colorectal cancer at a late stage, especially with metastases, significantly reduces patients’ chances of recovery [68].

Phenolic compounds, including XN, have anticarcinogenic effects in the development and treatment of cancer by reducing inflammation and antiproliferative activity, which translates into a reduction in the size of already existing cancerous tumors in the large intestine [69,70]. One of the main reasons for the development of cancer cells is access to energy material. Liu et al. [69] found that XN contributes to the suppression of AKT, thereby inhibiting the phosphorylation of hexoses by HKII (Hexokinase II), responsible for glycolysis, which is the main source of energy for colorectal cancer cells.

In vivo studies on mice have shown that the use of XN resulted in a reduction in cancer. The main reason for the slowdown of glycolysis is the ability of XN to inhibit EGFR (Epidermal Growth Factor Receptor), which activates the AKT kinase complex, whose AKT1 isoform participates in cell proliferation through protein synthesis, while the AKT2 isoform is responsible for metabolism, including glycolysis, thus contributing to cell apoptosis due to the lack of lactate and high pH [71]. In addition, apoptosis of cancer cells is enhanced by increased synthesis of pro-apoptotic Bax proteins (Bcl-2–associated X proteins) and reduced activity of anti-apoptotic Bcl-2 (B-cell lymphoma 2) proteins [72].

One of the standards for early detection of neoplastic lesions in the colon is the presence of apoptosis-resistant ACF (Aberrant Crypt Foci) formed before polyps [73]. XN has been shown to have an antagonistic effect on ACF, reducing the risk of developing colorectal cancer [74].

## 3. XN and Dysbiosis on the Gut–Brain Axis

### 3.1. Role of Gut Microbiota in the XN Demethylation Process

The formation of the intestinal microbiome as a result of antibiotic therapy, an unhealthy lifestyle and a given stage of life, and even the method of birth and lactation, may predispose to the development of food intolerances and allergies, gastrointestinal diseases, and even cancer [75,76,77]. The wide spectrum of phenolic compounds, especially XN and its metabolites, in the human body is an impulse for widely undertaken scientific research towards the prevention and treatment of various diseases.

The efficiency of XN depends on its transitions to its bioactive metabolites. Logan et al. [78], studying obese mice with impaired glycolipid metabolism, including a group lacking gut microbiota, found that regardless of the type of diet, with the addition of XN (low and high energy), improvements in parameters were observed only in mice with a naturally occurring gut microbiota. Referring to the described compound, it should be emphasized that XN and the microflora of the gastrointestinal tract have a direct effect, as it has a direct effect on bacteria, which at the same time, during its metabolization, partially transform it into other bioactive forms, such as 8-PN, where demethylation by CYP1A2 (Cytochrome P450 family 1 subfamily A member 1) occurs, followed by hydroxylation of CYP2C19 (Cytochrome P450 family 2 subfamily C member 19) and CYP2C8 (Cytochrome P450 family 2 subfamily C member 8), which leads to the formation of 8-PN [79,80].

It is worth noting that IXN and 8-PN are not the only bioactive metabolites of XN. Zang et al. [81] speculate that TXN (tetrahydro-XN) and α,β-dihydro-XN (DXN) are also important in the treatment of cardiovascular disease risk, type 2 diabetes mellitus, and metabolic syndrome in particular. They proved that TXN contributed to the expression of the gene responsible for the maintenance of inflammation by suppressing the gene responsible for the synthesis of IL-6 and Tnf-α in white adipose tissue and IL-22 (Interleukin 22) in the large intestine, which is responsible for inflammation in response to microbial infections. DXN supplementation, on the other hand, increased the concentration of occludin, a protein involved in the sealing of semi-permeable cell membranes. The improvement of these parameters is due to, among others, the observed increase in the number and diversity of intestinal bacteria of the genera Bacterioidetes and Tenericutes.

Finally, studies by Cermak et al. [82] have shown that the phenolic compounds and beta-acids present in hops, including those found in XN, exhibit strong antimicrobial activity. Notably, these compounds are effective against *Clostridium difficile*, a spore-forming bacterium that poses serious health risks, particularly after antibiotic therapy.

### 3.2. Effect of XN on Gut–Brain Axis Balance in Neurogenerative Disorders

The microbiota of the gastrointestinal tract refers to naturally occurring bacteria that live in symbiosis and commensalism in a healthy body. The basic functions of intestinal bacteria are the breakdown of food residues after digestion with enzymes, the synthesis of vitamins and short-chain FA, protection against pathogens, and communication with organs through the gut–brain axis, through the nervous, endocrine, and immune systems [83,84,85].

Including XN in the diet as a supplement can help not only in modulating inflammatory diseases but also in enhancing the quality of life by preventing mental clarity. XN achieves this by inhibiting the degradation processes in nerve cells. It contributes to reducing the concentration of Aβ (Amyloid β), a substance capable of crossing the blood–brain barrier, which can limit the progression of diseases such as Alzheimer’s disease. This effect suggests that cognitive functions may be improved with XN supplementation [86].

Sun et al. [87] confirmed XN’s efficacy in inhibiting the progression of Alzheimer’s disease by enriching the diets of mice with XN doses of 30 and 90 mg/kg body weight, significantly reducing proteins that accelerate apoptosis in the hippocampus. Moreover, Liu et al. [88] observed that one of the effects of XN supplementation in Alzheimer’s disease is a beneficial alteration in the gastrointestinal microbiota composition.

A positive correlation between antibiotic therapy and processes leading to the development of neurodegenerative diseases should be emphasized. Antibiotics can cause an imbalance in the gut–brain axis, which has been gaining importance in recent years. Dodiya et al. [89], implementing treatment of mice with a mixture of antibiotics (kanamycin, gentamicin, colistin, metronidazole, vancomycin), found that in males, Aβ deposition was inhibited along with a decrease in pro-inflammatory cytokines, and at the same time, poor gut microbiota was noted. In females, on the other hand, no inhibition of Aβ deposition was observed, but an increase in pro-inflammatory cytokines and a richer gut microbiota were observed.

To further confirm the role of the gut microbiota in the pathological deposition of Aβ, the authors performed an FMT (Fecal Microbiota Transplantation) from the mice were raised separately with ad libitum food and water access. Following the FMT, an increased rate of Aβ deposition and higher levels of pro-inflammatory cytokines were observed. This finding suggests that XN supplementation might be necessary to inhibit the progression of Alzheimer’s disease, especially in individuals predisposed to this condition.

## 4. XN as a Remedy for Menopause Symptoms and Bone Structure

### 4.1. Effects of XN on Postmenopausal Hormones

Menopause, which occurs as a result of ovarian failure to produce estrogens, contributes to a decrease in the quality of life of women. Women affected by inhibition or reduced production of the main female hormones struggle with many side effects, external ones affecting their self-esteem and internal ones contributing to the reduction in the efficiency of internal organs.

The first signs of menopause are hot flashes and cold sweats. They are caused by a sudden increase in peripheral blood pressure and the activation of sweat receptors to dissipate heat through the skin.

The most common method of treating menopause is undergoing hormone replacement therapy (ERT), but this has a number of side effects, such as liver strain, cardiovascular disease, and, in the case of long-term hormone therapy, even breast or endometrial cancer, which is associated with taking medications to counteract these changes [90,91].

One of the popular ERT alternatives that reduce menopausal symptoms and decrease the risk of several side effects is taking preparations containing XN, with a chemical structure similar to 17-β-estradiol [92,93]. The estrogenic activity of the prenylated XN metabolite results from O-demethylation occurring in the large intestine by G(+) of the *Eubacterium limosum* bacterium and by enzymes encoded by cytochrome P-450 genes (Cytochrome P-450), where demethylation occurs by CYP1A2, followed by hydroxylation of CYP2C19 and CYP2C8, which leads to the formation of 8-PN.

8-PN remains the strongest phytoestrogen to date, which, due to its similar structure to 17-β-estradiol, competes for the place of the estrogen receptor Erα (Estrogen receptor α) and ERβ (Estrogen receptor β) of the cell membrane, surpassing the biological activity of other known phytoestrogens, such as daidzein or genistein [94,95,96].

Kim et al. [97] found that feeding ovarian rats with a mixture of red clover and xanthohumol (in a 3:1 ratio) contributed to the reduction in hot flashes and also reduced the concentration of LDL cholesterol and triglycerides while causing an increase in the concentration of HDL (high-density lipoprotein) cholesterol. In addition, there was also an inhibition of weight gain, which was not observed in the group receiving only red clover extract.

In a study involving 120 women with early signs of menopause who were given hops extract in the form of tablets, a significant improvement in well-being was reported after just 4 weeks of the experiment compared to the placebo group. The authors concluded that the observed beneficial effect of hop extract, containing xanthohumol and its metabolites formed in the gastrointestinal tract, may be an alternative to ERT [98].

### 4.2. XN Reduces Oxidative Stress and Promotes Bone Cell Proliferation by Inhibiting Aβ

Estrogen deficiency is directly correlated with bone resorption, where binding to the estrogen receptor is followed by the expression of genes encoding proteins such as IGF-1 (Insulin-like growth factor), IL-1 (Interleukin 1), and TGF-β (Transforming growth factor β) while inhibiting bone resorption as a result of NF-κB inhibition [98,99].

Brier et al. [100] found the efficacy of XN in the treatment of osteoporotic lesions caused by Aβ, which causes a decrease in osteoblast proliferation and acceleration of cell apoptosis. As it was shown, XN contributed to the increase in the synthesis of Bcl-2 protein, thus inhibiting apoptosis. In addition, XN contributes to the reduction in ROS osteoblasts.

In another study, Xia et al. demonstrated that the use of XN in mouse nutrition effectively reduced the concentration of pro-inflammatory interleukins by increasing the concentration of SOD (Superoxide dismutase), which resulted in the breakdown of free radicals and OCN (Osteocalcin), which is necessary for bone mineralization [101].

On the other hand, Sun et al. [102], enriching the diet of mice with altered bone structure with hop extract with and without the addition of xanthohumol for 3 months, observed a significant improvement in the condition of the bone structure. There was an increase in the concentration of proteins involved in bone metabolism and cell proliferation with a simultaneous reduction in oxidative stress and activation of the gene encoding the RUNX2 (Runt-related transcription factor 2) protein.

One environmental factor contributing to osteoporosis is 2,3,7,8-tetrachlorodibenzo-p-dioxin (TCDD). Dobrzyński et al. [103] proved that anti-inflammatory compounds alleviate the effects of dioxins, which contribute to the deterioration of bone tissue. Similar conclusions were reached by Całkosińska et al. [104], establishing that pathological changes in mouse bone tissue resulting from reduced concentrations of calcium, magnesium, phosphorus, and iron induced by TCDD are reversible when their diet is enriched with XN.

Table 1 provides a concise summary of the XN antioxidant pathway and its role in the discussed diseases.

## 5. Challenges and Limitations of XN Applications for Disease Prevention

Xanthohumol (XN) is a notable phenolic compound widely appreciated in the cosmetics and dietary supplement industries for its antioxidant and anti-inflammatory properties [105]. Despite significant scientific interest, XN has not yet qualified as a drug, remaining classified as a dietary supplement. A primary factor limiting its progression to therapeutic use is the scarcity of in vivo studies, particularly in human subjects [106]. Existing research, mostly conducted on rodent models (Table 1), suggests that XN can mitigate disease symptoms by modulating cellular responses through the activation or inhibition of specific kinases that regulate gene expression. However, these studies often have limited experimental durations, leaving the long-term effects of XN unexplored [107].

One notable point highlighted in the manuscript is the ambivalent nature of XN’s biological activity. While XN has demonstrated numerous protective effects, there is some evidence suggesting that, under certain conditions, it may exhibit “dual-action” properties. This refers to the possibility that XN could have contrasting effects depending on the context, potentially influencing the progression of specific cell types, such as prostate cancer and glioma cells. Although such risks are not the primary focus of this review, they underscore the need for caution and further investigation into the mechanisms behind XN’s varied effects.

Even if it has been proven that there are no undesirable side effects with a high supply of XN, it should be remembered that this compound is not bioavailable in itself [108] because the biologically active molecules are its metabolites formed in the gastrointestinal tract by demethylation resulting in the formation of IXH, 6-PN, 8-PN, TXN, or DXN [109,110]. There is still no effective method to determine the exact half-life of XN and its metabolites in blood serum [111]. What is known, however, is that a significant part of the ingested XN is excreted unchanged in the feces and urine. This may be due to two different pathways: the low solubility of XN, which translates into equally low bioavailability in the lumen of the gastrointestinal tract, and, on the other hand, the ability of XN to attach to proteins in the cytosol. Moreover, the non-polar predominance of the XN structure affects the change in its spatial structure after attachment to phosphatidylcholine occurring on the surface of cell membranes [112,113].

Although XN has shown potential in alleviating symptoms and supporting conventional treatments, its long-term efficacy and safety remain unverified, emphasizing the need for further research to establish XN’s potential as a therapeutic compound.

## 6. Conclusions

This review emphasizes the antioxidative properties of xanthohumol (XN) as a central feature in its potential for supporting health, particularly in the context of modern diseases. XN’s strong antioxidant effects play a significant role in reducing oxidative stress, a contributor to inflammation and disease progression in conditions such as large intestine tumors, gastric ulcers, and neurodegenerative disorders. While conventional therapies often involve drawbacks like drug resistance and chemotherapy-related side effects, XN presents a promising alternative due to its antioxidative action and low toxicity, allowing it to be administered in varying doses without histopathological damage.

Research suggests that XN’s antioxidative mechanisms also interact with cellular pathways linked to energy regulation and autophagy, especially in malignant tumors. Although this dual role has the potential to disrupt energy balance within cancer cells, it also highlights the complexity of XN’s effects in cancer treatment, particularly in cancers such as prostate and glioma, where antioxidative and metabolic actions could both suppress and inadvertently support tumor cell survival. Although these findings are primarily based on animal studies, they provide a strong foundation for future human research focused on XN’s antioxidative benefits and its implications for nutrition-based approaches to disease prevention.

## Figures and Tables

**Table 1:** Antioxidant Effect of Xanthohumol in Disease Prevention. Pictographs show the effects: increase ↑, decrease ↓.

Organ/System	Disease/Disorder	Model	Pathway	Action	Results	Reference
Liver	Fibrosis	Mice	TGF-β1/NF-κB	↓ NF-κB inhibit synthesis of MCP-1, ICAM-1	↓ Inflammation, ↑ Inactive form of stellate cells, collagenase synthesis	Kisseleva et al. [53] Lee et al. [40]
Pancreas	Cancer	Mice	NF-κB/TNF-α/IL-6/IL-17/mTOR/AKT	↓ NF-κB/TNF-α/IL-6/IL-17/mTOR/AKT/VEGF, ROS	↓ Inflammation, ROS, ↓ VEGF, IL-8 ↑ NrF-2, IL-10, ARE	Huangfu et al. [32] Vazquez-Cervantes et al. [33] Silke et al. [39]
Colon	Cancer	Mice	EGFR/AKT/ACF/HKII/	↑Inhibition of EGFR ↓ ACT and HKII activity, ↑ Bax synthesis, ↓ ACF	↓ inflammation, polyps formation, glycolysis ↑ cancer cells apoptosis	Liu et al. [69] Guo et al. [72]
	Microbiome	Mice	XN/P450/ IXN/TXN/DXN	↓ IL-6/Tnf-α/IL-22, ↑ occludin	↓ inflammation, C. difficile activity ↑ mucosa tight junction, Bacterioidetes and Tenericutes	Cermak et al. [82] Zhang et al. [81]
Stomach	Ulcers	Rats/in vitro	Bcl-2/Bax, NF-κB/ROS	↓ Blc-2, NF-κB ↑ Bax, ROS	↓ inflammation ↑ cancer cells apoptosis	Minaiyan et al. [23] Wei et al. [24]
Nervous system	Alzheimer	Mice	mTOR/LC3II, Bax/Bcl-2	↓ IL-6, Interleukin-1β	↓ Aβ, Pro-inflammatory cytokines, ↑ autophagy, anti-apoptotic activity, ↑ cognitive functions	Sun et al. [87] Liu et al. [86]
Endocrine system	Menopause	Human/rats	O-demethylation/P-450	↑ ER-α, ER-β binding	↓ LDL, TAG, cholesterol ↑ HDL ↑ well-being	Kim et al. [97] Aghamiri et al. [98]
	Osteoporosis	Mice	TNF/IL-1/IL-6	↓ Nrf2, HO-1, IL-1, IL-6, Aβ, ↑ RUNX2	↑ anti-inflammatory cytokines, ↑ osteoblastic differentiation	Xia et al. [101] Sun et al. [102]
